# Genomic Characteristics and Pathogenicity of Novel Reassortant Mammalian Orthoreoviruses From Sheep, China

**DOI:** 10.1155/tbed/6025244

**Published:** 2025-05-15

**Authors:** Xia Li, Xuhang Cai, Yi He, Wenliang Li, Junjun Zhai, Runbo Luo, Sizhu Suolang, Li Mao, Bin Li

**Affiliations:** ^1^Institute of Veterinary Medicine, Jiangsu Academy of Agricultural Sciences, Nanjing, China; ^2^Key Laboratory of Veterinary Biological Engineering and Technology, Ministry of Agriculture and Rural Affairs, Nanjing, China; ^3^National Center for Engineering Research of Veterinary Bio-Products, Jiangsu Academy of Agricultural Sciences, Nanjing, China; ^4^Jiangsu Key Laboratory for Food Quality and Safety-State Key Laboratory Cultivation Base of Ministry of Science and Technology, Jiangsu Academy of Agricultural Sciences, Nanjing, China; ^5^Xizang Agriculture and Animal Husbandry University, Linzhi, China; ^6^Jiangsu Co-Innovation Center for Prevention and Control of Important Animal Infectious Diseases and Zoonoses, Yangzhou University, Yangzhou, China; ^7^Agriculture Development Service Center, People's Government of Chengguan Town, Fukang, China; ^8^Shaanxi Province Engineering and Technology Research Center of Cashmere Goat, Yulin University, Yulin, China

**Keywords:** mammalian orthoreoviruses (MRVs), pathogenicity, reassortment, sheep, virus isolation and identification

## Abstract

Mammalian orthoreoviruses (MRVs) have a wide geographic distribution worldwide and have been detected from humans and a variety of animal species. This study represents the first isolation of MRV from sheep rectal swabs in China, with analyses of its molecular and pathogenicity characteristics. MRV-positive samples were inoculated into Madin–Darby bovine kidney (MDBK) cells, resulting in stable cytopathic effects (CPEs) after three generations of blind passage. Two isolates were isolated and confirmed as MRV, named MRV-XJ23 and MRV-sheep/SY13, through reverse-transcription polymerase chain reaction (RT-PCR), transmission electron microscopy, and indirect immunofluorescence assay (IFA). The viruses exhibited broad cellular tropism. Whole-genome sequences were obtained and subjected to homology and evolutionary analyses, revealing that MRV-XJ23 and MRV-sheep/SY13 belong to the MRV-1 serotype. Phylogenetic analyses demonstrated that MRV-XJ23 is a reassortant virus containing gene segments from three MRVs that infected humans, bovines, and bats, with nucleotide homology exceeding 94.56%. The gene segments of MRV-sheep/SY13 were derived from five strains—Osaka2005, BatMRV-2/SNU1/Korea/2021, T1/human/Netherlands/1/84, IND/MZ/3013814/reo, and B/03—with nucleotide homology exceeding 95.47%. Animal experiments demonstrated that MRV-sheep/SY13 infection induced significant pathological changes in the respiratory and digestive tracts of mice. In sheep, MRV-sheep/SY13 caused respiratory infections, but no obvious lesion was observed from the digestive tract. This study expands our understanding of the MRV host range, reveals the potential public health risk of MRV transmission across species and zoonotic transmission, and underscores the necessity of further studies on epidemiology, reassortment patterns, and pathogenicity of MRV in sheep and domestic animals.

## 1. Background

Mammalian orthoreovirus (MRV) belongs to the genus *Orthoreovirus* in the *Reoviridae* family, which is a nonenveloped virus with 10 segmented double-stranded RNA and divided into three size classes. There are three large segments (L1, L2, and L3), three medium segments (M1, M2, and M3), and four small segments (S1, S2, S3, and S4) [1, 2]. The S1 gene encodes two proteins, *δ*1 and *δ*1s. As a structural protein, the *δ*1 protein governs MRV serotype specificity and is directly involved in cell attachment, receptor binding, and neutralization by type-specific antisera. Based on neutralization assay and hemagglutination inhibition (HI) test targeting the *δ*1 protein, MRV is classified into four established serotypes: Lang-T1L (MRV-1), Jones-T2J (MRV-2), Dearing-T2D (MRV-3), Ndelle-T4N (MRV-4), and a putative MRV-5 [1, 3–5]. The *δ*1 protein exhibits serotype-specific variations, including amino acid deletions and insertions that lead to marked divergence in the homology of S1 sequences. This divergence enables the use of S1 sequences for genotyping through phylogenetic reconstruction, with results fully consistent with serotyping based on neutralization and HI assays [6].

Due to the apparent absence of species barriers, MRVs can infect a wide range of mammalian species, including humans, livestock, companion animals, and wild animals, such as pigs, sheep, cattle, cats, dogs, monkeys, mink, mice, and bats [6–10]. Despite the wide geographical distribution and host range of MRV, not all virus types cause severe diseases in mammals. To date, these viruses are primarily associated with mild gastroenteritis and respiratory disease and may be symptomatic or asymptomatic in infected animals. In Malaysia, MRVs have been discovered in patients suffering from acute respiratory diseases and have shown the risk for human-to-human transmission [11, 12]. MRV-3 is associated with diverse clinical manifestations in pigs, including fever, diarrhea, and respiratory diseases, with cases reported in Europe, Asia, and North America [13–16]. Reoviruses have also been implicated as pathogens inducing neurological symptoms. Necrotizing encephalopathy and meningitis caused by MRV-2 or MRV-3 have been reported in infected humans in Europe and the United States [10, 17]. Additionally, central nervous system symptoms have been observed in mice infected with MRV-1 or MRV-3 [2, 18, 19]. In China, MRV-2 was isolated in Sichuan and found to infect neonatal piglets, causing severe diarrhea [20].

The segmented nature of the MRV genome can lead to the formation of new recombinant viruses with unpredictable biological properties, thus posing a risk for animals and human beings. Because of the segmented characteristics of MRVs, when multiple MRV lineages infect the same host, reassortments can occur, driving viral evolution and generating new strains [7]. Reassortant MRVs were isolated in several species, including voles, partridges, bats, and calves. Based on the genome sequences and evolutionary analyses, ample evidence has been provided that these MRV gene segments may be derived from different hosts and regions [21–23]. For example, a novel MRV strain named SI-MRV01, which showed more than 97% identity with MRVs from bats of *Pipistrellus* spp. and *Myotis* spp., was identified in a child with gastroenteritis, red and swollen gums, and oral ulcers [24–26]. Another reassortant MRV strain from piglets of China was analyzed, and its segments were originated from MRVs of humans, pigs, chamois, bats, and mink [20]. In Italy, two novel reassortant swine MRVs were identified on the same farm, sharing similar genetic backbone, and reassortment was present in the S1, S4, and M2 segments from pig, bat, and human MRVs as well as other unknown sources [27]. In the United States, a reassortant MRV containing segments from three MRV serotypes infecting humans, bovines, and swine was identified in pigs with ~40% mortality [28]. These findings highlight the possibility of cross-species transmission between MRVs. In this current study, two sheep-derived MRV strains were isolated from Jiangsu and Xinjiang. Sequence and phylogenetic analyses showed that these isolates shared high homology with MRVs from cattle, pigs, monkeys, bats, and humans and were classified as MRV serotype 1. Further animal studies demonstrated that these MRV isolates are capable of infecting and causing respiratory and digestive tract infections in mice and sheep.

## 2. Materials and Methods

### 2.1. Virus Isolation and Preparation

MRV-positive rectal swabs were collected from sheep suffering diarrhea in Xinjiang Uygur autonomous region and Jiangsu province. The MRV-positive samples were diluted with sterile phosphate-buffered saline (PBS) and then centrifuged at 12,000 rpm for 10 min. The supernatant was filtered through a 0.22-μm filter and inoculated onto a monolayer of Madin–Darby bovine kidney (MDBK) cells, cultured in Dulbecco's Modified Eagle Medium (Shanghai BasalMedia Technologies Co., Ltd., Shanghai, China) with 10% fetal bovine serum (Yeasen Biotechnology [Shanghai] Co., Ltd., Shanghai, China), at ~80% confluency. Cytopathic effects (CPEs) were observed every 12 h and subjected to three blind passages. Total RNA (Vazyme, Nanjing, China) was extracted from cell cultures exhibiting CPE and sent for polymerase chain reaction (PCR) assays and sequencing (Tsingke Biotech Co., Ltd., Nanjing, China). The median tissue culture infectious dose (TCID_50_) was detected by the Karber formula.

### 2.2. PCR and qPCR Detection Methods

The PCR assays were performed according to the instructions of the Green Taq Mix (Vazyme, Nanjing, China), The MRV genes were amplified by specific primers (Table 1). Briefly, the PCR mixture (25 μL) was prepared as follows: 1 μL of cDNA, 1 μL each of forward and reverse primers (10 μM), 12.5 μL of 2× Master Mix, and 9.5 μL of nuclease-free water. Amplification involved 95°C for 5 min, denaturation at 95°C for 30 s, annealing at 55°C for 1 min, and extension at 72°C for 1 min, with 35 cycles for each program from denaturation to extension, and a final extension at 72°C for 5 min in an automated thermal cycler (BIO-RAD, CA, USA). The PCR products were recycled and ligated to pMD-18-T plasmid (Takara), and positive plasmids were sequencing (Tsingke Biotechnology Co., Ltd., Nanjing, China).

The qPCR methods were performed according to the HiScript II One Step qRT-PCR Probe Kit kit instructions (Vazyme, Nanjing, China), as described before [29], which included 100 nM of each primer MRV-qL1F/R and MRV-probe (Table 1), 4 μL of RNA template, and deionized distilled water. The reaction conditions were reverse transcription at 50°C for 5 min, initial denaturation at 95°C for 30 s, 40 cycles at 95°C for 10 s, and final extension at 60°C for 30 s. The sensitivity was up to 10 copies/mL [23].

### 2.3. Electron Microscopy Analysis

MDBK cells were infected with the isolated MRV, and the cell cultures were harvested at 96 h postinfection. The supernatants were collected and concentrated by ultracentrifugation to purify MRV particles. Briefly, the cell cultures were subjected to three freeze–thaw cycles and centrifugated at 10,000 rpm for 30 min, followed by concentration at 35,000 × *g* for 30 min and 120,000 × *g* for 2 h in a Beckman Optima L-100XP ultracentrifuge with a 70 Ti rotor. The supernatant was discarded, and the sediment was resuspended in electron microscopy fixative. For negative staining, 10 μL of the virus sample was attached on a Formvar carbon-coated grid, stained by 3% phosphotungstic acid (pH 6.3) for 30 s, and inactivated with ultraviolet irradiation. The stained grids were air-dried, and virus particles were visualized using a transmission electron microscope (Hitachi HT7800; Hitachi, Tokyo, Japan).

### 2.4. Indirect Immunofluorescence Assay (IFA)

An IFA was used to detect MRV particles. In brief, the MRV-infected and control cells were fixed and permeabilized with 4% paraformaldehyde and blocked by 1% bovine serum albumin for 1 h. Then, the cells were incubated with rabbit anti-MRV polyclonal antibody (1:1000, prepared and stored in our lab) and were subsequently washed and stained with fluorescein isothiocyanate-labeled goat anti-rabbit antibody (1:100; BOSTER Biological Technology Co., Ltd., Wuhan, China) and 4′,6-diamidino-2-phenylindole (DAPI, 1:10,000; Beyotime, Shanghai, China). Observation and image acquisition were performed using an inverted fluorescence microscope.

### 2.5. Genome Sequencing and Phylogenetic Analysis

RNA extraction was performed following the manufacturer's instructions (Vazyme). First-round reverse-transcription PCR (RT-PCR) for RNA viruses was conducted using HiFiScript gDNA Removal RT MasterMix (CWBIO, Nanjing, China), followed by second-round PCR with Green Taq Mix (Vazyme). Each run included a positive PCR control containing DNA amplicon and nuclease-free water as a negative control. PCR products were visualized on 1% agarose gels, cloned into the pMD-18T vector (Takara, Kusatsu, Japan), and sent to Tsingke Biotechnology (Beijing, China) for sequencing.

The whole genome of the virus (L1–S4) was amplified from the viral genomic RNA with specific primers (Tsingke Biotechnology) (Table 1). Each segment sequence of the isolate was BLASTed against the existing sequence in GenBank to record the best identity hit of each gene (Supporting Information 1: Table S1). Sequences of closely related and representative MRV strains were downloaded for phylogenetic analysis. Based on the open reading frame, MEGA 11 (https://www.megasoftware.net/) is used to build a neighbor-joining tree for each segment with the neighbor-joining method and a bootstrap value of 1000 replications. The pairwise genetic distance was generated by Megalign software and refined as a heatmap using an online tool (https://www.chiplot.online/tvbot.html).

### 2.6. Cell Tropism Experiment

The cell tropism of MRV was assessed using human colorectal cancer cells (HRT-18), monkey kidney cells (MA104), Vero cells, F81 cells, porcine intestinal epithelial cells (IPEC-J2), and MDBK cells. All cell lines were obtained from ATCC and stored in our lab. MRV, bovine viral diarrhea virus (BVDV), and mycoplasma were detected as negative before used. The anti-MRV polyclonal antibody from rabbit was employed in IFA to identify viral particles between different cell types.

### 2.7. Animal Infection Study Design

All animal studies were conducted with approval from the Animal Research Ethics Committee of the Jiangsu Academy of Agricultural Sciences. All animals were clinically healthy and confirmed serologically and virologically negative for MRV prior to the experiments.

#### 2.7.1. Mouse Infection

Two groups of BALB/c mice were administered intraperitoneal injections of 100 μL or nasal drops of 50 μL of MRV-XJ23 (TCID_50_ = 10^5^/0.1 mL) or cell culture medium, with five 2-week-old mice in each group. Fecal samples were collected at 4- and 7-days postinfection (dpi), and the viral load in the samples was measured. The L1 gene was also amplified and sequenced using RT-PCR. The mice in each group were euthanized at 10 dpi for further pathological analysis. In necropsy, tissue lesions were evaluated, and tissues, including the intestinal tract, lungs, kidneys, heart, liver, and spleen, were collected for histopathological analyses and virological detection.

#### 2.7.2. Sheep Infection

To further assess the pathogenicity of isolated MRV, animal experiments were conducted on sheep. Nasal, throat, and anal swabs and sera samples were collected from sheep prior to the experiment and sent for diarrhea pathogens detection, such as MRV, cpCoV, CEV, AstV, and AdV by RT-PCR/PCR. Sheep were tested negative for the above pathogens and used for subsequent pathogenicity experiments [29]. Six 2-month-old healthy sheep were randomly divided into two groups. One group received 4 mL of MRV-XJ23 (TCID_50_ = 10^5^/0.1 mL) by nasal injection, and the other received 4 mL of medium. Body temperature and clinical symptoms were monitored daily. Nasal, throat, and rectal swabs were collected daily, and blood samples were obtained at 0, 3, 5, 7, 9, 11, and 13 dpi. At the endpoint of 14 dpi, all animals were euthanized by humane methods with intramuscular injection of Telazol and intravenous injection of 10% KCl, as described before [29]. MRV in the collected samples was detected using the qRT-PCR method as described before [23]. The L1 gene was also amplified and sequenced using RT-PCR. All sheep were euthanized at 14 dpi, and tissue lesions were evaluated and recorded. Tissues of heart, lungs, trachea, liver, spleen, kidney, duodenum, jejunum, and ileum were collected for virological detection and histopathological analyses. Viral load in the collected samples was quantified using fluorescence, and the tissues were histopathologically examined after fixation and hematoxylin and eosin (HE) staining.

## 3. Results

### 3.1. Two MRV Strains Were Isolated From Clinical Samples

**Figure 1:**
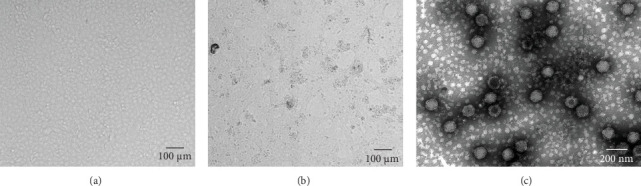
CPE of MRV-XJ23 infection. (A) Control MDBK cells incubated with PBS. (B) CPE was observed in MDBK cells incubated with MRV. (C) Electron micrograph of MRV particles. CPE, cytopathic effect; MDBK, Madin–Darby bovine kidney; MRV, mammalian orthoreovirus; PBS, phosphate-buffered saline.

MRV-positive rectal swabs were processed and inoculated into MDBK cells. At 72 h post inoculation, characteristic CPE, including cell contraction and roundness, reticular lesions, and shedding, was observed, while the negative control cells remained normal (Figure 1, Supporting Information 2: Figure S1). Two MRV strains, designated MRV-XJ23 and MRV-sheep/SY13, were successfully isolated from the Xinjiang uygur autonomous region and Jiangsu province, respectively. Virus isolation was confirmed by RT-PCR and sequencing targeting the L1 gene, while negative results were obtained for other viral families. The viral titers of MRV-XJ23 and MRV-sheep/SY13 were determined to be 10^5^ TCID_50_/0.1 mL and 10^4.5^ TCID_50_/0.1 mL, respectively.

The virus particles were purified by ultracentrifugation and negatively stained with 2% phosphotungstic acid. Double-layered, nonenveloped, and icosahedral viral particles with diameters of ~70–80 nm were clearly observed by transmission electron microscopy, exhibiting the typical morphological features of MRV (Figure 1, Supporting Information 2: Figure S1). IFA using rabbit anti-MRV polyclonal antibody demonstrated distinct fluorescence signals in MRV-infected MDBK cells, whereas no signal was observed in uninfected controls. These results confirmed the successful isolation of sheep-derived MRV.

### 3.2. The Novel MRVs Infected in Different Cell Lines

**Figure 2:**
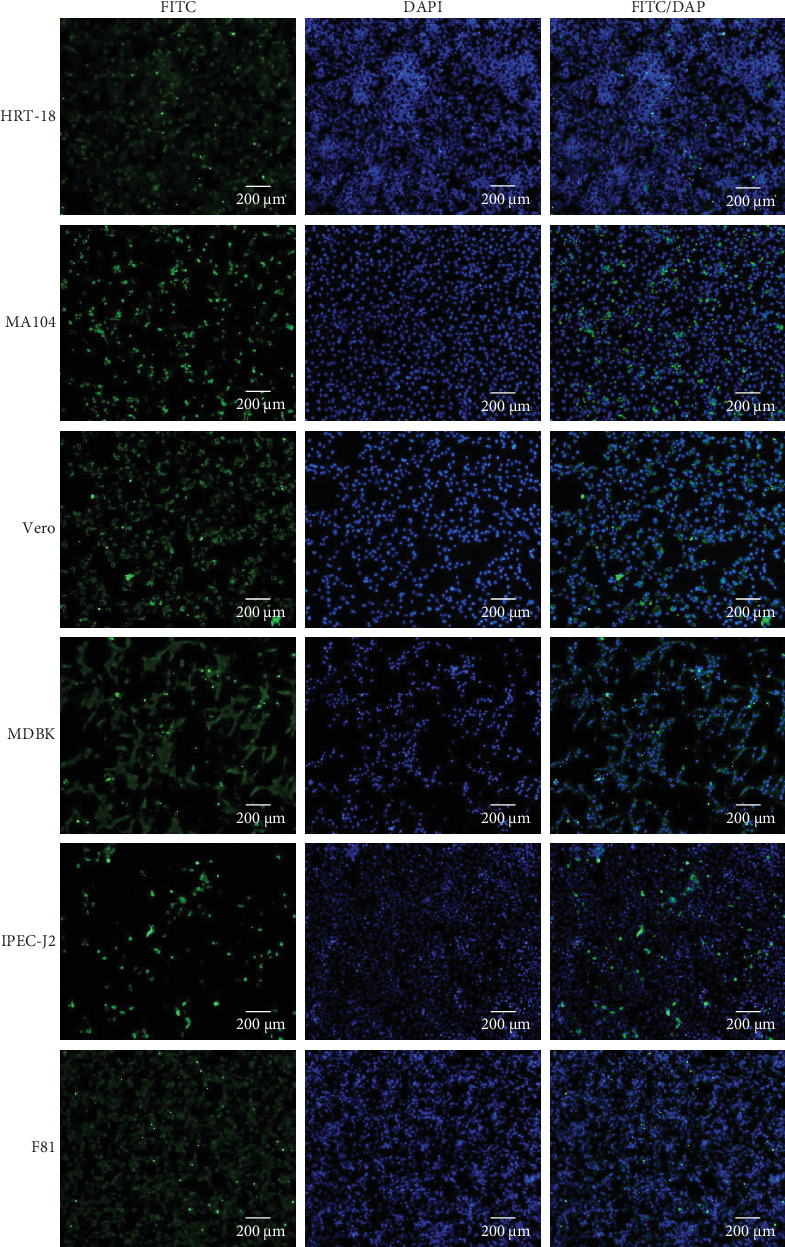
MRVs exhibit broad cell tropism. Three days postinfection with MRV-XJ23, HRT-18, MA104, Vero, F81, IPEC-J2, and MDBK cells were subjected to indirect immunofluorescence analysis. Sheep MRV antigens were stained with rabbit anti-MRV polyclonal antibody as the primary antibody, followed by fluorescein isothiocyanate-labeled mouse anti-rabbit IgG (green). Cell nuclei were counterstained with DAPI (blue). Scale bar: 200 μm. DAPI, 4′,6-diamidino-2-phenylindole; MDBK, Madin–Darby bovine kidney; MRVs, mammalian orthoreoviruses.

A wide range of cell lines from cattle, monkeys, cats, pigs, and humans were sent to assess the tropism of the MRV isolates. All tested cells were susceptible and permissive for MRV, exhibiting obvious CPE and positive results in IFA, which indicates a potentially broad host range for the virus (Figure 2, Supporting Information 2: Figure S2).

### 3.3. Full Genome Sequence and Phylogenetic Analysis of the MRVs

To explore the evolutionary relationship between the isolated MRV strains and reference reoviruses, the complete genomes of all 10 segments were obtained and compared with closely related strains. The GenBank accession numbers for each segment of MRV-XJ23 were PP235840–PP235849, and those for MRV-Sheep/SY13 were PP942556.1–PP942565.1.

**Figure 3:**
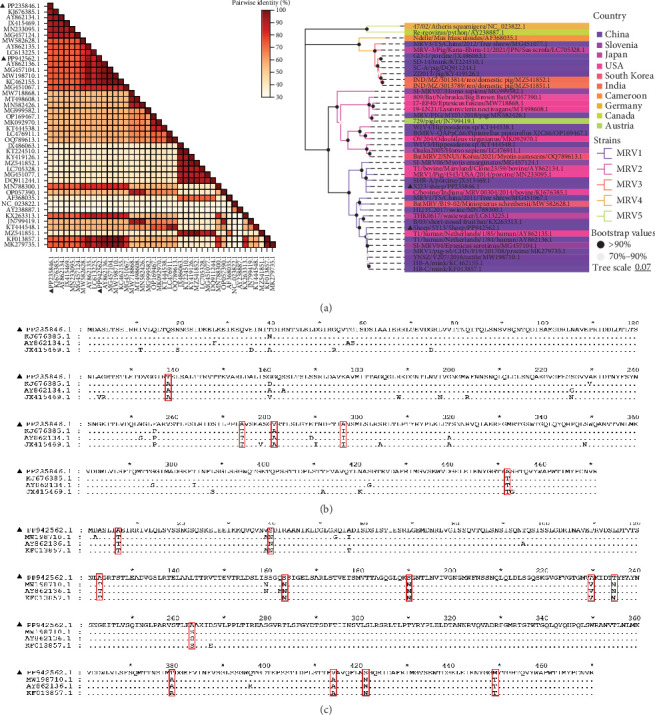
Phylogenetic and genomic analyses of MRV-XJ23 and MRV-sheep/SY13. (A) Phylogenetic tree and pairwise genetic distance heatmap of MRV-XJ23 and MRV-sheep/SY13 based on the complete nucleotide coding sequences of the S1 fragments. The tree was constructed using the neighbor-joining method in MEGA 11 with bootstrap values from 1000 replicates and was refined using https://www.chiplot.online/tvbot.html. Different MRV lineages and countries of origin are indicated by color blocks, and MRV-XJ23 and MRV-sheep/SY13 are marked with black triangles. The pairwise genetic distance heatmap was created using Megalign software. (B and C) Alignment of deduced amino acid sequences of the *δ*1 protein. Comparison of the *δ*1 protein sequences from MRV-XJ23, MRV-sheep/SY13, and closely related MRV-1 strains. (B) Alignment for MRV-XJ23. (C) Alignment for MRV-sheep/SY13. MRV, mammalian orthoreovirus.

The S1 gene encodes *δ*1 protein, which determines the serotype of MRV. Sequence comparison analysis showed that both MRV-XJ23 and MRV-sheep/SY13 belong to the MRV-1 serotype. The S1 gene of MRV-sheep/SY13 shared 95.47% nucleotide homology with the Netherlands human MRV-1 isolate T1/human/Netherlands/1/84, while the S1 segment of MRV-XJ23 displayed 94.56% homology with an MRV-1 isolate (C/bovine/Indiana/MRV00304/2014) detected in calves from the United States in 2014. Phylogenetic analysis indicated that MRV-XJ23 and MRV-sheep/SY13 belong to the same evolutionary branch as MRV-1 isolates from humans, minks, cattle, and pigs, clustering together on the genetic evolutionary tree (Figure 3A). MRV-XJ23 showed the closest relationship with the bovine-derived isolate C/bovine/Indiana/MRV00304/2014, while MRV-sheep/SY13 was most closely related to the anthropogenic isolate T1/human/Netherlands/1/85. The results confirmed that MRV-XJ23 and MRV-sheep/SY13 belong to the MRV-1 serotype, though the two isolates occupy different branches of the genetic evolution. The *δ*1 protein, encoded by the S1 gene, mediates virus attachment [30]. Comparison of *δ*1 protein sequences from MRV-1 strains available in GenBank revealed that MRV-XJ23's *δ*1 protein was comprised of 471 amino acids, with five substitutions identified in conserved regions (138T, 255F, 274A, 281V, and 452A), while MRV-sheep/SY13's *δ*1 protein comprised of 470 amino acids, with 12 substitutions in conserved regions (7A, 40S, 123A, 163S, 190S, 230A, 235T, 263A, 379T, 414V, 421S, and 449M) (Figure 3B,C).

**Figure 4:**
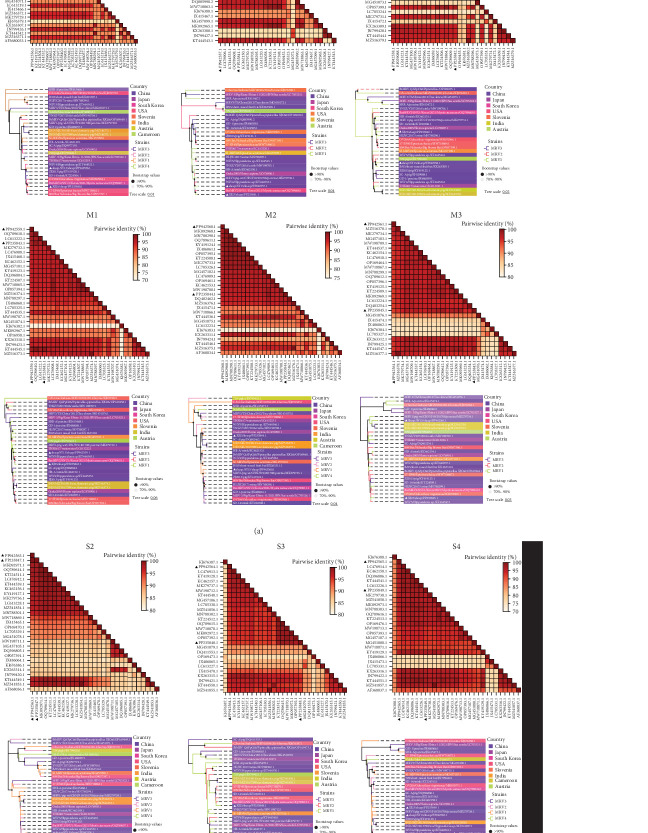
Phylogenetic and genomic analyses of MRV-XJ23 and MRV-sheep/SY13. Phylogenetic tree and pairwise genetic distance heatmap of MRV-XJ23 and MRV-sheep/SY13 based on the complete nucleotide coding sequences of the S2–S4, M1–M3, and L1–L3 segments. The tree was constructed using the neighbor-joining method in MEGA 11 with bootstrap values from 1000 replicates and was refined using https://www.chiplot.online/tvbot.html. Different MRV lineages and countries of origin are indicated by color blocks, and MRV-XJ23 and MRV-sheep/SY13 are marked with black triangles. The pairwise genetic distance heatmap was created using Megalign software. MRV, mammalian orthoreovirus.

**Figure 5:**
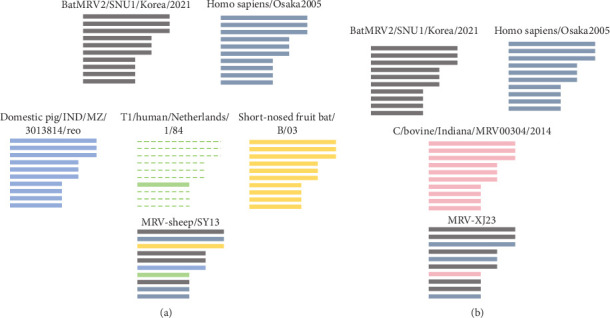
Schematic summary of the genomic compositions of novel MRV isolates and related strains. Segments with close relationships were identified based on nucleotide identity and phylogenetic analysis, with each distinguished by a unique color. Dashed lines indicate unidentified segments of the T1/human/Netherlands/1/84 strain. (A) Indicates MRV-sheep/SY13 strain. (B) Indicates MRV-XJ23 strain. MRV, mammalian orthoreovirus.

The L1, L2, M1, M3, S2, and S3 segments of MRV-XJ23 shared a close relationship with the strain BatMRV-2/SNU1/Korea/2021 (98.15%–99.14% nucleotide homology), while the remaining three segments showed high homology with the *Homo sapiens* MRV-2 (Osaka2005) strain detected in Japan. Sequence analysis predicted five strains as the parental strains of MRV-sheep/SY13. The Osaka2005 strain from Japan, originating from *H. sapiens*, contributed to the reassortment of the L2, S3, and L4 segments of MRV-sheep/SY13 (97.09%–98.18% nucleotide homology). Similarly, the BatMRV-2/SNU1/Korea/2021 strain from Korea contributed to the reassortment of the L1, M1, M2, and S2 segments of MRV-sheep/SY13 (97.60%–98.25% nucleotide homology). Notably, the IND/MZ/3013814/reo strain from a domestic pig in India participated in the reassortment of the M3 segment with MRV-sheep/SY13 (97.18% nucleotide homology), while the China B/03 strain reassorted with MRV-sheep/SY13 in the L3 segment (98.48% nucleotide homology) (Figures 4 and 5). This study revealed that the genetic fragments of two sheep-derived MRVs were clustered together with those of other animal origins, including that sheep-derived MRV fragments do not form a specific branch but often exchange genetic material with viruses from other sources.

### 3.4. Pathogenicity of MRV in Mice and Sheep

**Figure 6:**
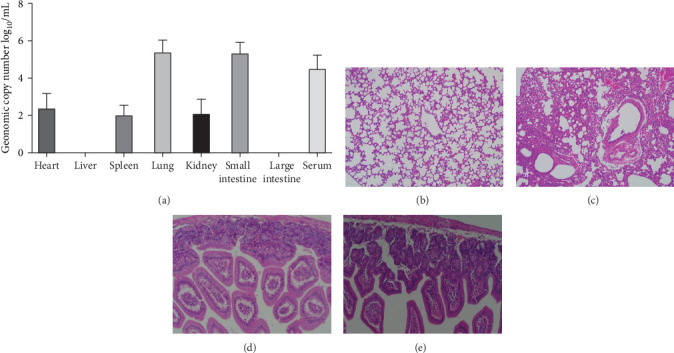
Tissue analysis and pathological examination of MRV-infected mice. (A) Genome copy number of MRV in various mouse tissues. (B–E) HE staining results. (B) Lungs of the control group. (C) Lungs of the infected group. (D) Small intestine of the control group. (E) Small intestine of the infected group. HE, hematoxylin and eosin; MRV, mammalian orthoreovirus.

Pathogenicity of MRV in mice. To evaluate the MRV pathogenicity, experiments were conducted on mice. No significant clinical symptoms, such as diarrhea, acute gastroenteritis, or central nervous system disease, were observed in the mice, and MRV was not detected in stool samples. The virus replicated effectively in the lungs, small intestine, and serum, while low viral loads were detected in the heart, spleen, and kidneys. The L1 gene was also analyzed using RT-PCR and sequencing from the samples, the results showed the MRV-XJ23 replicated in the sheep and shed it outward through excretions. MRV was not detected in the liver or large intestine (Figure 6A). HE staining revealed no significant pathological changes in the heart, liver, spleen, kidneys, or large intestine of infected mice. However, infected mice exhibited narrowed or absent alveoli, lymphocytic infiltration in the lungs, submucosal congestion, and partial lymphocytic infiltration in the small intestine (Figure 6). RT-PCR results from control mice were negative, and no significant abnormalities were observed in their HE results. These findings suggest that MRV infection induces respiratory and intestinal lesions in BALB/c mice.

**Figure 7:**
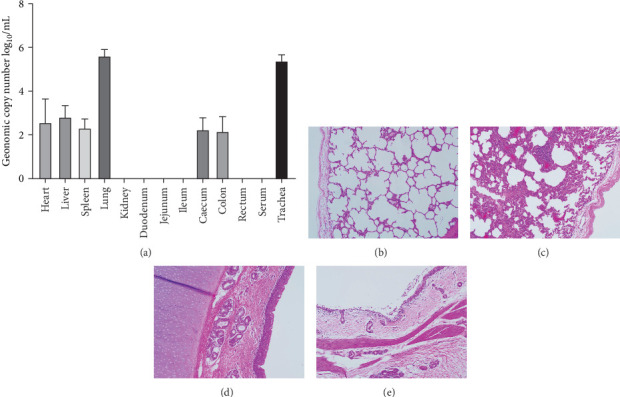
Tissue analysis and pathological examination of MRV-infected sheep. (A) Genome copy number of MRV in various sheep tissues. (B–E) HE staining results. (B) Lungs of the control group. (C) Lungs of the infected group. (D) Trachea of the control group. (E) Trachea of the infected group. HE, hematoxylin and eosin; MRV, mammalian orthoreovirus.

Pathogenicity of MRV in sheep. Following MRV infection, in two of the sheep, the body temperature transiently increased to 40.1°C at 3–4 dpi and then returned to normal, and rhinorrhea and occasional cough were observed during this period, whereas the control group showed no such symptoms. The qRT-PCR test detected MRV in one sheep's throat swab at 3 dpi and nasal swab at 4 dpi. All animals were euthanized at 14 dpi for analysis, and high viral loads in the lungs (10^5.62^ genome copies/mL) and trachea (10^5.39^ genome copies/mL) were detected, while low viral loads were observed in the heart, liver, spleen, cecum, and colon (10^2.17^–10^2.83^ genome copies/mL). The L1 gene was also amplified and sequenced as MRV-XJ23. No virus was detected in the remaining samples (Figure 7A). To evaluate general pathological effects, the heart, liver, lungs, intestine, spleen, and other tissues were subjected to histopathological examination. Samples were paraffin-embedded, sectioned, and stained with HE. Infected sheep exhibited thickened alveolar walls, lymphocytic infiltration, diffuse interstitial pneumonia, and extensive necrosis and detachment of pseudostratified ciliated columnar epithelium (Figure 7D,E).

## 4. Discussion

MRV has a wide range of hosts and can infect a variety of mammals, such as humans, bats, cattle, monkeys, sheep, swine, rodents, snakes, baboons, and birds [6, 8–10, 28, 31]. Animals infected with MRV may exhibit overt symptoms or remain asymptomatic. The MRV-3 strain, for example, was reportedly isolated from the lungs of a healthy Italian alpine rock antelope that displayed no clinical signs or lung lesions [32, 33]. In China, MRV-3 has been identified in the feces of diarrheic pigs and healthy sloths, while MRV-1 has been detected in the feces of symptomatic sloths and tissue samples of wild short-nosed fruit bats [34–36]. In our laboratory, MRV was first detected in diarrheic goats using quantitative RT-PCR [23]. As one of the largest global producers and consumers of mutton, China has faced an increased risk of pathogen transmission due to large-scale and intensive farming practices. Such conditions facilitate the potential for cross-species transmission of MRV from sheep to humans or other animals through direct contact or environmental vectors, posing significant challenges to outbreak containment.

In this study, two strains of MRV were isolated from geographically distant regions, Xinjiang and Jiangsu. The viruses exhibited a wide range of cellular adaptations and were capable of replicating efficiently in various mammalian cell lines, including Vero, MDBK, MA104, and HRT-18. Although host tropism was assessed at the cellular level, this assessment may not fully reflect natural infections in human beings or other animals. Nevertheless, the potential for cross-species infections posed by these novel viruses warrants scientific attention [2, 37].

The S1 gene encodes the *δ*1 protein and determines the serotype of MRV. Sequence and phylogenetic analyses of the S1 gene revealed that both isolates belong to the MRV-1 serotype, but they are located in different evolutionary branches. The S1 genes of MRV-sheep/SY13 and MRV-XJ23 encode 470 and 471 amino acids, respectively, with amino acid substitutions in certain conserved regions. These substitutions might alter the structure and function of the *δ*1 protein, potentially affecting the pathogenicity of the viruses and posing a threat to humans and other mammals.

The segmented nature of MRV facilitates recombination between strains from different sources, leading to the generation of new viral strains. In this study, the MRV-XJ23 isolate was predicted to have arisen from reassortment involving three parental strains: BatMRV-2/SNU1/Korea/2021, Osaka2005, and C/bovine/Indiana/MRV00304/2014. By contrast, the MRV-Sheep/SY13 isolate was generated through the reassortment of five parental virulent strains: BatMRV-2/SNU1/Korea/2021, Osaka2005, IND/MZ/3013814/reo, B/03, and T1/human/Netherlands/1/84. Both isolates shared BatMRV-2/SNU1/Korea/2021 and Osaka2005 as parental strains, suggesting that these two strains may be widely prevalent in the region and frequently undergo reassortment with different strains. Moreover, the S2 gene sequences of the isolates were 100% identical despite being from geographically distant regions, indicating that reassortment also occurs between sheep isolates. These findings suggest that MRV reassortment occurs across different regions, serotypes, and hosts. Reports of reassortant MRV isolations from animals such as bats, pigs, and rock antelopes further highlight the prevalence of MRV rearrangements between different hosts [22, 32, 34]. However, the mechanisms underlying the generation of novel viruses and the potential role of intermediate hosts remain unknown and warrant further investigation [32]. The parental strains of MRV-Sheep/SY13 include human-derived MRV. In the phylogeny of the human-derived TO-151/BR strain, the S1 gene is closely related to a porcine Asian strain, while the other segments show a closer relationship with the MRV-3 strains from different geographical positions and hosts, including humans and bats [38]. Porcine-derived MRV-2 is a novel reassortant strain involving human, porcine, muntjac, bat, and mink MRVs, as shown by nucleotide identity and phylogenetic analysis [20]. Similarly, two MRV strains isolated from pig farms in northeastern Italy showed another evidence of reassortment. The M2, S1, and S4 genes of these isolates displayed distinct characteristics, likely obtained from reassortment with MRVs originating from bats, humans, or other unidentified hosts [27]. Furthermore, three of the six bat-derived MRV strains were found to share high similarities in the S1 segment with MRVs isolated from piglets, minks, or humans [39]. Literature has also documented that recombination and reassortment can occur between animal-derived and human-derived MRVs without significant interspecies barriers, highlighting the potential for such events to alter many viral characteristics. These changes pose substantial challenges to the prevention and control of MRV epidemics worldwide [23]. This study emphasizes the importance of ongoing MRV surveillance and genomic characterization analysis from sheep to better understand the evolution, epidemiology, and zoonotic potential [40].

Recently, several studies have shown that MRV can lead to serious illness and even death in humans and animals, primarily manifesting as respiratory infections, diarrhea, and encephalitis. However, most individuals infected with MRV experience no significant clinical signs or only mild respiratory or digestive tract symptoms [31]. MRV-1 has been found to infect mink with no obvious symptoms, though some cases exhibited mild diarrhea, whereas infected dogs suffered severe diarrhea and, in some cases, death [6]. MRV-2 caused mild lung damage in infected nursing mice and replicated in multiple organs for 3 weeks [41]. Studies in pigs showed that directly exposed pigs showed symptoms of fever, diarrhea, and runny nose [7]. Pathogenicity analysis showed that although bovine MRV had no clinical symptoms after infecting mice, it caused damage to the intestinal, lung, liver, kidney, and brain [42]. Other research demonstrated that MRV-1 induces respiratory disease in BALB/c mice following nasal inoculation [36]. By contrast, the present study showed that nasal and oral administration of MRV-1 induced both respiratory and digestive tract infections in mice, leading to significant pathological changes in these systems. The clinical symptoms and pathological changes observed in mice varied depending on the viral dose and the pathogenicity of the viral strains. Animal regression experiments were conducted to further investigate the pathogenicity of different reassortant isolates. In sheep, MRV causes respiratory and digestive tract infections, with pathological changes predominantly observed in the respiratory tract. Although the virus was isolated from anal swabs of diarrheic animals, it was not detected in fecal samples during animal regression experiments and did not cause significant gastrointestinal lesions. Nevertheless, detailed information about the pathogenicity of the isolates, the mechanisms underlying their pathogenicity, and their potential for zoonotic transmission requires further investigation.

Gene rearrangements, an important mode of RNA virus mutation, may lead to changes in viral pathogenicity [43]. Avian and human influenza viruses undergo rearrangements in swine to generate new viruses with the ability to spread across hosts [44]. In influenza viruses, gene rearrangement has been shown to be one of the important causes of the emergence and epidemiology of new strains. The viral genomes of the 1957 Asian influenza and the 1968 Hong Kong influenza pandemic both contained gene fragments from influenza viruses of both avian and human origins, confirming the epidemiological significance of cross-species gene rearrangement [45]. The segmented genome structure of MRV gives it an enhanced potential for gene rearrangement. This suggests the need to pay close attention to MRV genetic variation, especially gene rearrangement, in order to detect new viral strains and assess their potential pathogenicity in a timely manner to avoid serious harm to the livestock industry.

## 5. Conclusions

We isolated and characterized reassortant MRV strains from sheep, which were derived from MRVs of different host origins, including human-origin MRV. The isolates were capable of infecting both mice and sheep, causing respiratory disease. Our findings indicate that MRV not only poses a significant threat to ovine health but also exhibits zoonotic potential, capable of cross-species and transboundary dissemination. This poses new challenges to animal disease prevention and control as well as public health in China's border areas and customs. Therefore, continued surveillance and pathogenicity studies of MRV in sheep are essential to better understand the viral pathogenicity, transmissibility, and mutation dynamics, thus helping to prevent the emergence of new mutated MRVs that may threaten both animal and public health.

## Figures and Tables

**Table 1:** Primers for MRV amplification.

Primer name	Sequence 5′- 3′	Fragment size (bp)
MRV-F	TTCACTCAGGCATTATCCGA	559
MRV-R	TCCGCTTCTGACTCCTGA	
cpCoV-N-F	AAGGTGTGCCTATTGCACCAG	500
cpCoV-N-R	GCTTAGTTACTTGCTGTGGC	
CEV 5′-UTR-F	CTTTGCACGCCTGTTTTCC	497
CEV 5′-UTR-R	CACACGCTCGGAGGTTGGGAT	
BoAstV-DP-F	GAYTGGACBCGHTWTGATGG	418
BoAstV-DP-R	KYTTRACCCACATNCCAA	
BAdV-pVIII-F	TCAGGACCGCCTGGATCATA	796
BAdV-pVIII-R	TCAGCCACGCAAAGCCATTT	
MRVL1-F	GCTACACGTTCCACGACAATGTCA	3854
MRVL1-R	GATGAGTTRACGCRCCACGRC	
MRVL2-F	GCTATTGGCGCRATGGCGAAC	3915
MRVL2-R	GATGAATTAGGCRCGCTYACGA	
MRVL3-F	GCTAATCGTCAGGATGAAGCGGA	3901
MRVL3-R	GATGARTCGGCCCAACTAGCATY	
MRVM1-F	GCTATTCGCGGTCATGGCTTACAT	2304
MRVM1-R	GATGAAGCGCGTACGTAGTCYTAG	
MRVM2-F	GCTAATCTGCTGACCGTYACTCT	2203
MRVM2-R	GATGATKTGCCTGCATCCCTTAACC	
MRVM3-F	ATGGCTTCATTCAAGGGAT	2166
MRVM3-R	TTACAGCTCATCRGTTGGAAC	
MRVS1-F	GCTATTCGCGCCTATGGATGCR	1466
MRVS1-R	GATGATTGACCCCTTGTGCCGA	
MRVS2-F	GCTATTCGCTGGTCAGTTATGGCT	1331
MRVS2-R	GATGAATGTGTGGTCAGTCGYGAG	
MRVS3-F	GCTAAAKTCACGCCTGTYGTCGT	1198
MRVS3-R	GATGATTAGGCGYCACCCACCA	
MRVS4-F	GCTATTTTTGCCTCTTCCYAAACG	1196
MRVS4-R	GATGAATGRAGCCTGTCCCACGT	
MRV-qL1F	GTCTCAGGCTCGACAGATTAAG	—
MRV-qL1R	TGCAGAACGGGATCATATAAGG	
MRV-probe	FAM-AGTTTGTGTTGGCGTTATTGGTGGC-TAMRA	

## Data Availability

All the data are contained within the article and the Supporting Information.

## Supporting Information

Supporting Information 1 Table S1. Reference strains used in phylogenetic tree and pairwise genetic distance heatmap ADDIN NE.Bib.

Supporting Information 2 Figure S1. CPE of MRV-sheep/SY13 infection. (A) Control MDBK cells incubated with PBS. (B) CPE was observed in MDBK cells. Figure S2. MRVs exhibit broad cell tropism. Three days postinfection with MRV-sheep/SY13, HRT-18, MA104, Vero, F81, IPEC-J2, and MDBK cells were subjected to indirect immunofluorescence analysis. Sheep MRV antigens were stained with rabbit anti-MRV polyclonal antibody as the primary antibody, followed by fluorescein isothiocyanate-labeled mouse anti-rabbit IgG (green). Cell nuclei were stained with DAPI (blue). Scale bar: 200 μm.
